# Supplementary material for: Analysis of proteome adaptation reveals a key role of the bacterial envelope in starvation survival
Source: Mol Syst Biol. 2022 Dec 7;18(12):e11160. doi: 10.15252/msb.202211160 (PMC9728487; doi:10.15252/msb.202211160)
Supplement: Supplementary file 1 — Expanded View Figures PDF [file MSB-18-e11160-s003.pdf]

Expanded View Figures

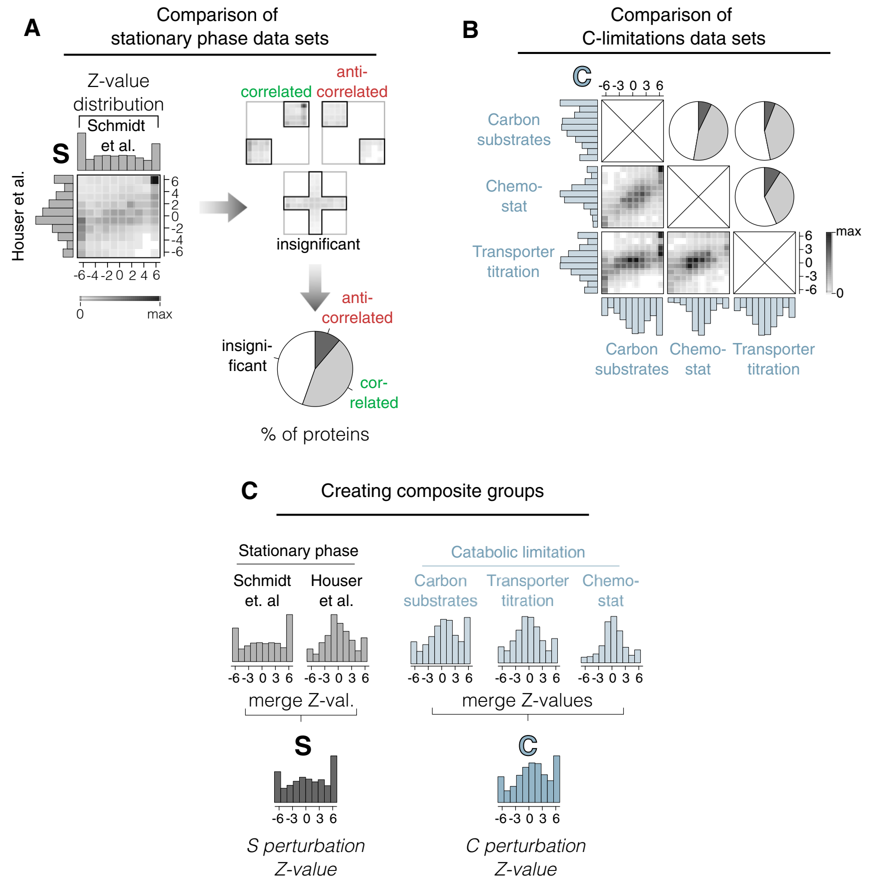

Figure EV1. Comparison and merging of MS proteomics data from different sources.

A Comparison of *Z*-value distributions from two different data sets, by Houser *et al* (Data ref: Houser *et al*, 2015b) and Schmidt *et al* (Data ref: Schmidt *et al*, 2016b), both measured in stationary phase after growth on glucose. On left: *Z*-value distributions of individual experiments are shown on left and top of correlogram. Shaded area inside the correlogram depicts the frequency distribution of individual proteins that are measured in both data sets. On right: Proteins scored in top right and bottom left corner are counted as "correlated," and proteins in the top left and bottom right are counted as "uncorrelated." Cut-off for significance is chosen at $Z = 1.28$, corresponding to a *P*-value of 0.1. Proteins with at least one *Z*-value less than 1.28 are counted as uncorrelated. On bottom: Quantitative analysis of correlation. Only a minority of proteins are observed to anticorrelate between data sets, with the majority either correlating or being statistically insignificant.

B Analysis of correlograms and quantification in pie charts of three data sets of different catabolic limitation analog to panel A. "Carbon substrates" and "chemostat" taken from Schmidt *et al* (Data ref: Schmidt *et al*, 2016b), "Transporter titration" taken from Hui *et al* (Data ref: Hui *et al*, 2015b). Different types of catabolic limitation show a high correlation between data sets.

C Data sets from panels A and B, respectively, are merged to form a single Z-value distribution for each growth perturbation.

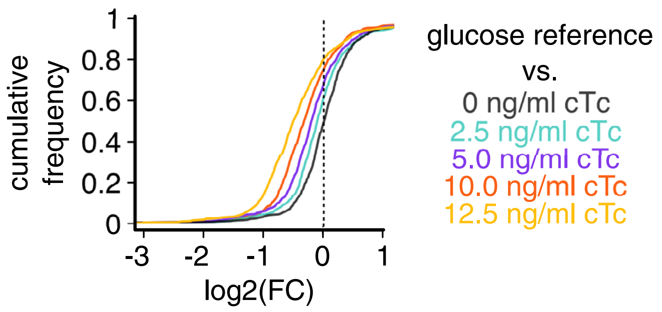

Figure EV2. Cumulative distribution of fold changes under expression of a useless protein (O condition).

Fold change of protein abundances (FC) in LacZ overexpression using strain NQ1389 compared with the reference condition of wild-type *Escherichia coli* grown on glucose. The abundance of the vast majority of proteins decreases with the induction of LacZ (inducer: chlortetracycline, cTc).

 ©2022 The Authors

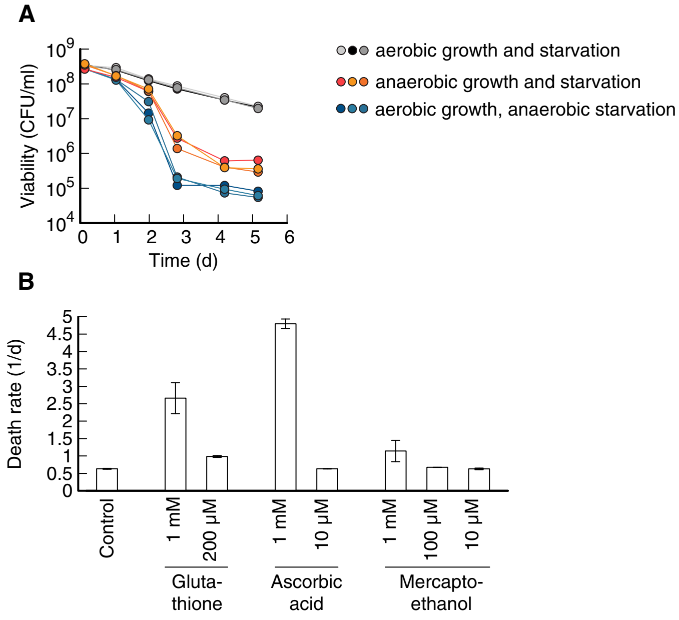

Figure EV3. Starvation in the absence of oxidative damage.

A Viability over time in *Escherichia coli* cultures starved in either anaerobic or aerobic conditions. Cultures were previously grown on a glucose minimal medium. Neither performing both starvation and growth (orange), nor only starvation in anaerobic conditions (blue) decreased death rate. Instead in both cases, viability decreased several orders of magnitude more compared with aerobic conditions. This finding is in disagreement with previous claims that anaerobic starvation prevents loss of viability (Dukan & Nyström, 1999) but matches the observation that bacteria actively recycle nutrients from dead cells (Schink *et al*, 2019), which is more efficient if bacteria can use respiration.

B Starvation in the presence of antioxidants glutathione, ascorbic acid (Vitamin C), and beta-mercaptothanol. High concentrations of antioxidants increase the death rate, while low concentrations show no significant effect over control. No antioxidant condition significantly decreased the death rate. Three biological replicates per condition. Error bars show mean ± standard deviation.

Source data are available online for this figure.

© 2022 The Authors

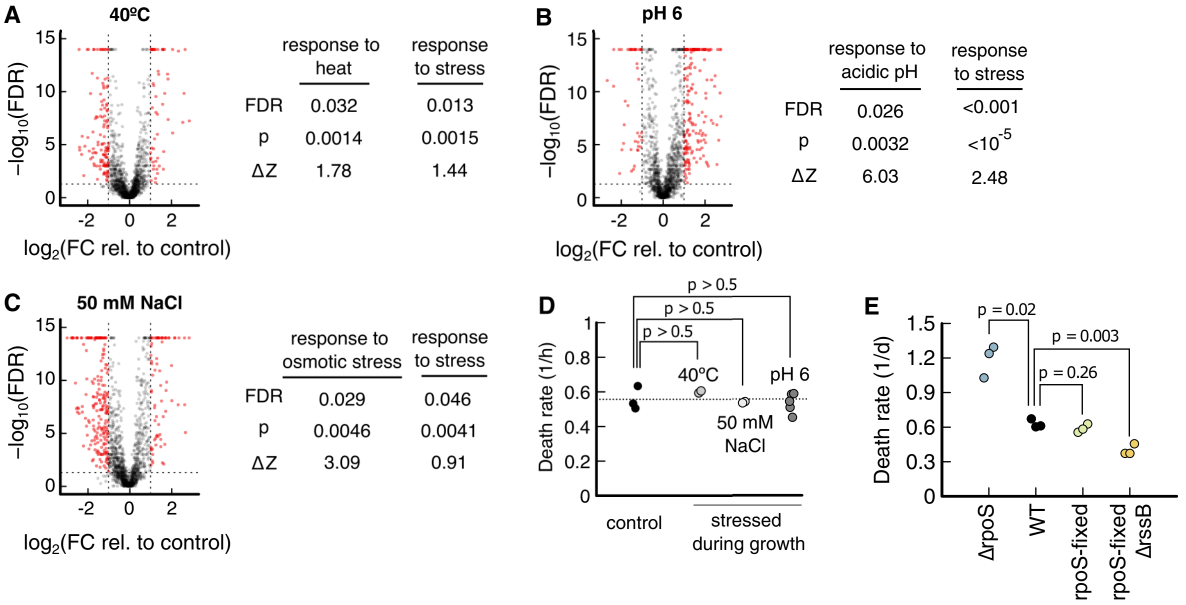

Figure EV4. Effect of prestressing on proteome and survival kinetics.

A–C Comparison of stress conditions to a glucose reference. On left side of each panel, volcano plots of individual proteins, showing the probability of being a response versus the logarithm of the fold change. Proteins with a fold change higher than 2, i.e., $\log_2(2) = 1$, and false discovery rates smaller than 0.05, i.e., $\log_{10}(0.05) = -1.3$, are colored in red. On the right side of the panel, for each prestress (40°C, pH 6, and 50 mM NaCl), the corresponding stress response and the general "response to stress" are tested for significant upregulation using Kolmogorov–Smirnov tests. In each prestress, both the specific and the general stress response are significantly upregulated, FDR < 0.05. Data source: (Data ref: Schmidt *et al*, 2016b).

D After stressing during growth, bacteria are transferred to prewarmed, carbon-free minimal medium without stress. Death rates of neither prestressing condition led to a significant change in death rate, $P > 0.5$. Number of biological replicates: Control (3), 40°C (2), NaCl (2), and pH 6 (5).

E Death rate of wild-type NCM3722 compared with ΔrpoS, a "rpoS-fixed" strain where an amber stop codon in *rpoS* is restored (Mori *et al*, 2021) and a strain with "rpoS-fixed" and ΔrssB. All mutants have NCM3722 as background. Three biological replicates per condition.

Data information: *P*-values in panels D and E are calculated using a two-tailed *t*-test.
Source data are available online for this figure.

 ©2022 The Authors

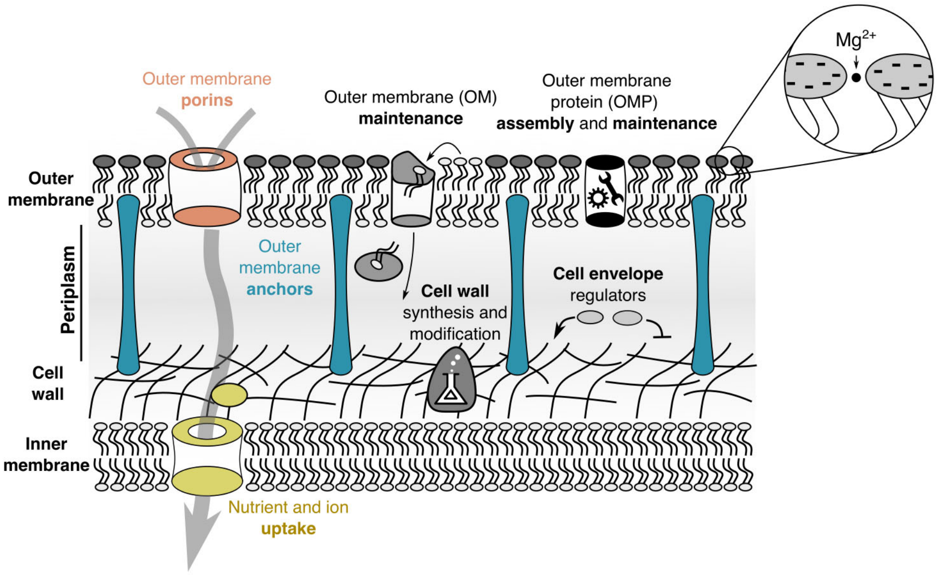

Figure EV5. The cell envelope of *Escherichia coli*.

For gram-negative bacteria, the cell envelope consists of the outer membrane, the cell wall, and space between the inner and outer membrane called periplasm. Proteins in the cell envelope have diverse functions. Outer membrane anchors (blue), specifically Lpp and OmpA, connect the outer membrane to the cell wall. Outer membrane porins (red) are large tunnel-shaped proteins that facilitate the diffusion of biomolecules and ions across the outer membrane. Nutrient uptake systems (yellow) import biomolecules and ions across the inner membrane. While most of the nutrient uptake system is in the inner membrane, thus not considered as part of the envelope, binding proteins (yellow circle in periplasm) are. There are over 100 different kinds of binding proteins in *E. coli*, which make up a significant fraction of the proteome of the cell envelope. Other proteins include proteins involved in the maintenance of the outer membrane, e.g., the Mla system which shuffles phospholipids from the outer leaflet of the outer membrane to the inner membrane. Outer membrane protein (OMP) assembly and maintenance are proteins that fold outer membrane proteins, e.g., Bam complex, proteases, which degrade misfolded proteins, e.g., DegP, and chaperones that prevent folding of outer membrane proteins in solution, e.g., Skp. Cell envelope regulators include the CpxAR signal transduction system and regulation of RpoE via anti-sigma factor RseA. Cell wall synthesis and modification include cell wall hydrolases and regulators of cell wall synthesis. Lipid A, the lipid on the outer leaflet of the outer membrane is highly charged and requires Mg^{2+} ions for shielding (Schneck *et al*, 2010).

© 2022 The Authors
