# Supplementary material for: Analysis of proteome adaptation reveals a key role of the bacterial envelope in starvation survival
Source: Mol Syst Biol. 2022 Dec 7;18(12):e11160. doi: 10.15252/msb.202211160 (PMC9728487; doi:10.15252/msb.202211160)
Supplement: Supplementary file 12 [file MSB-18-e11160-s009.pdf]

molecular systems biology

Analysis of proteome adaptation reveals a key role of the bacterial envelope in starvation survival

Severin Schink, Constantin Ammar, Yu-Fang Chang, Ralf Zimmer, and Markus Basan
DOI: 10.15252/msb.202211160

Corresponding author(s): Markus Basan (markus@hms.harvard.edu) , Severin Schink (info@severin-schink.eu)

Review Timeline:

Submission Date:	1st Jun 22
Editorial Decision:	11th Jul 22
Revision Received:	15th Sep 22
Editorial Decision:	18th Oct 22
Revision Received:	19th Oct 22
Accepted:	19th Oct 22

Editor: Jingyi Hou

Transaction Report:

(Note: With the exception of the correction of typographical or spelling errors that could be a source of ambiguity, letters and reports are not edited. Depending on transfer agreements, referee reports obtained elsewhere may or may not be included in this compilation. Referee reports are anonymous unless the Referee chooses to sign their reports.)

1st Editorial Decision **11th Jul 2022**

Thank you for submitting your work to Molecular Systems Biology. We have now heard back from the three reviewers who agreed to evaluate your manuscript. As you will see from the reports below, the reviewers acknowledge the interest of the study. They raise, however, a series of concerns, which we would ask you to address in a major revision.

I think the reviewers' recommendations are relatively straightforward, and there is no need to reiterate all the points listed below. All issues raised by the reviewers need to be satisfactorily addressed. As you may already know, our editorial policy allows in principle a single round of major revision, and it is therefore essential to respond to the reviewers' comments that are as complete as possible.

On a more editorial level, we would ask you to address the following issues:

Reviewer #1:

Overall comment:
I have read the manuscript entitled "Adaptation of bacterial proteome reveals a key role of the cell envelope in starvation survival" authored by Schink et al. This is an interesting study that analyzes multiple protein abundance datasets across conditions and identified membrane-related genes play a key role to prevent cell death. The authors studied the trade-offs between fast growth and long survival and ranked more than 2000 E. coli proteins that are related to survival genes.

Interestingly, they found the most important survival genes are playing roles in the maintenance of envelope integrity during starvation. These discoveries provide growing evidence of how the outer membrane is essential for the survival of non-sporulating bacteria. However, this manuscript needs significant work, specifically around the methods utilized, addressing the specific comments outlined below.

Major comments:
1. CACe-score is not a very clear name for this quantity - and it is not explicitly defined in the Methods, only in Figure S1. Recommend making sure that this specific term appears somewhere in the methods, and recommend changing the name of this score to something less unwieldy. Possibility: "Survival score". The name for this score doesn't have to precisely capture the underlying mathematical procedure used to compute it, as long as that procedure is very clear in the Methods and the term is defined explicitly there.
2. The authors should carefully provide supplementary files to include raw data (e.g., protein abundance in the investigated conditions). The current pdf for review only has scattered tables, which are not readable. I found them from Biorxiv (10.1101/2022.05.18.492425) but it seems that only processed data (i.e., averaged values without error ranges) is available and raw data is still missing.
3. Since method and raw data were not sufficiently provided, it is difficult to understand how genes were chosen for testing in Figure 2. I see that some genes have high positive CACe-score values even if they have N/A or negative values in some conditions. And why do they fluctuate depending on conditions? Also, some of the tested genes have a vCACe-score value of nearly zero (e.g., bamE). Please elaborate on how the major finding is associated with the main analysis.
4. Similarly, the major conclusions are based on the death rate under different conditions. Please elaborate on the method about how to calculate the death rate was calculated in the Method section.
5. The presence/absence of the "O" in "CARLOS" is confusing throughout. For example, in Figure S1, the top-right header implies that "O" is included in the CACe-score, while the histograms in the bottom center imply that it is not (which indeed it isn't). Consider eliminating "O" from the acronym and treating it as more of an "add-on" than an integral part of the survival score.

Minor comments:
Abstract: "...to narrow down the set of proteins that..."
Abstract: "...are related to the cell envelope..."
Introduction: "...the only energy source for heterotrophic..."
Introduction: "number of active processes without losing their viability" (remove hyphen)
Results: Figure 1B: 0 to max range in the color bar - what is max? Difficult to interpret 2-D histograms if they are all scaled differently. And it is not also clear what each gray square means and how the value was calculated. In the caption of Fig 1, some of the citations are referring to Fig. S3, not Fig. S2.
Results: Figure 1C: FDR - is this just p-value? FDR is the threshold set for p-value correction, but I'm not sure that the corrected p-value itself can be interpreted as the actual false discovery rate. Consider re-labeling this column to "corrected p-value" or similar.
Results: "proteome changes in the remaining CARLS conditions are partially independent of each other, as evidenced by a significant fraction of Z-values being anticorrelated in each comparison" - what statistical test was used to determine the significance of the anti-correlated Z-values?
Results: "We next wanted to test if any..."
Results: "...in anaerobic conditions, where oxidative damage should be absent" (remove "the rate")
Results: "and up to 23% for bacteria..." (remove "a staggering")
Results: "...belonging to stress response and oxidative damage, showed little..." (add comma)
Figure 2A, please indicate both growth stage and medium for each condition.
Figure S2: "less than X" - what is X?
Figure S3: y-axis should be 0 to 1 for the cumulative frequency label (similar to Figure 1C). The 0 and 2.5 colored lines are not clearly visible, making interpretation difficult.
Figure S5: legend says proteins with FC higher than 2 are red, but plot has red color for proteins with FC greater than 1
Figure S5: what does the bracket between 40C and pH 6 mean?
Results: "...mechanical integrity of the cell envelope plays a causal role..." (remove "is")
Results: "supplementing starved culture with EDTA" - Figure 2D has DNP on the x-axis - which is correct?
It seems that a death rate is referred for both "cell" and "protein". Please differentiate them in the text and detail how they were calculated if not mentioned clearly.
Figure 1 panel A "Glucose minmal medium" should be "Glucose minimal medium";
Figure 1C, the author emphasized that several GO-processes were enriched, including "Response to oxidative stress" and "Response to stress". It seems that the "Response to stress" means non-oxidative stress. It is necessary to appropriately describe the "Response to stress" here.
Each growth condition has a corresponding death rate. Assuming that the authors tested the death rate under the glucose minimal conditions. It should be noted in the figure legend.
Please check and appropriately make the font italic. Here Line 140, Biselli et al., 2020; Line142, and other lines.
Line 163 "rseA" should be "RseA"
Line 178 "is plays a causal" should be "plays..."

Reviewer #2:

A better understanding cell death represents a unique opportunity in bacterial physiology. Previous work by the authors established key mechanisms underlying the death rate in E. coli K-12: 1. balance between maintenance rate and recycling yield sets the death rate, and 2. That there is a trade-off between growing faster and surviving longer. This study is a natural follow-up and addresses what the molecular determinants of adaptation are during this growth-death transition.

The authors compare mass spectrometry (MS) data for different growth perturbation experiments (CARLOS) to obtain candidate proteins by scoring their correlation strength across datasets. Upon further annotation proteins involved in the cell envelope functions show a considerable increase in death rates. This is confirmed by a genetic screen where the knockouts display elevated death rates. Next, the involvement of these mutants in impairing cell wall integrity and prior knowledge of the effects of ion homeostasis on death leads the authors to hypothesize that the integrity of cell wall in general and membrane permeability, in particular, is crucial and is more specifically mediated by the cost of ion homeostasis.

To test this hypothesis they minimize ion diffusion across the membrane by using an osmo-balanced medium to find a decrease in death rate and that the growth-death dependence across different conditions is absent. If the cost of ion homeostasis determines membrane integrity, even for widely different investments into the cell envelope proteins for different conditions, we would expect similar death rates, hence the observation (Fig. 3). This supports their hypothesis and leads them to conclude that the growth-death rate dependence from Fig 1A follows from differences in cell envelope integrity under those conditions.

We feel that this finding is novel and represents a significant advance that the readers of MSB would be interested in. Overall, we recommend this work for publication after the authors respond to the questions and comments below.

Major points:

The manuscript was quite hard to follow around the statistical scoring part. I feel parts of Fig S2 should be included in the main text or some more time should be spent on explaining the entirety of Fig. 1. Another suggestion is to include an illustration that runs through calculations and scores in Fig. 1b by using an example for 1 protein.

Line 206: While Fig 3. is the culmination of this story, it does merit a more elaborate explanation, in that it was not obvious why the authors would expect the tradeoff to go away upon using an osmo-balanced medium. Maybe elaborating this inference could help make the expectation clearer.

How do the authors decide to wash the medium rather than let the nutrients deplete naturally? I am ok with the experiments being in some sense "unnatural", but I also feel that it would be good for the reader to be aware of which conclusions depend on this and which do not.

Minor points:

Introduction:

1st and 2nd para:
"This finite...": What does this refer to? Aren't both the aforementioned resources finite?
The first and second paragraphs can be demarcated better. "...But what determines..." could be shifted to the second paragraph.

Results:
Para 4: line 98: "...as evidenced by a significant...": Does anti-correlation imply independence?

Grammatical errors/typos:

Line 17: set of proteins*
Line 116: wanted TO test
Line 126: rate oxidative?
Line 178: extra "is"
Line 196: Add citation
Line 207: reduce*
Line 243: stabilize

Reviewer #3:

The manuscript by Schink and colleagues investigates how bacteria reorganize the proteome to promote starvation survival. Using a set of 6 growth perturbations ("CARLOS" conditions, defined in Figure 1, drawing on previously published data) to generate an ensemble of proteome responses to nutrient limitations, they identify a set of candidate proteins linked with survival and lifespan-and in doing so evade the challenges often associated with this task, namely the fact that most physiological measures can be correlated with lifespan. To do so, they calculate a statistical score ("Z-value") that quantifies the probability that protein abundance is correlated (or anti-) with longer survival. They calculate regularized Z-values for each dataset and then aggregated Z-values per condition. When compared pairwise, the Z-values per condition show enough variation (e.g. sizable fraction of Z-values are anti-correlated, yet Z-values for "similar" perturbations are correlated) that they are assumed partially independent, which is the key to identifying a reduced set of candidate proteins linked with starvation survival. The authors then narrowed their focus, guided by experiments, to a set of cell envelope proteins, and they found that knocking out these envelope proteins-which span multiple functions (e.g. cell wall synthesis or cell envelope regulation)-significantly modulated starvation survival (porins are a notable exception). They complemented these findings by applying antimicrobials targeting the cell envelope, which they show kill the cells during starvation (a notable corroborating finding given that many antibiotics, such as beta-lactams, kill in a growth-rate dependent manner and would lose effectiveness in starvation conditions). Finally, they show that the death-rate dependence is eliminated in osmotically balanced media, suggesting (along with previous work) a connection to ion homeostasis.

Overall, I find the work interesting and timely. It draws on a clever use of previous data to identify target proteins linked with survival, and importantly, tests those predictions experimentally for a collection of proteins. The results are surprising given that the periplasm and outer membrane are typically considered to function primarily as barriers to entry (for, say, antimicrobials or other harmful substances), and I therefore believe it would interest a broad range of readers. I have only the following minor comments for the author to consider:

-- The paper does a nice job of extracting simple principles (i.e. cell envelope integrity modulates starvation survival, perhaps driven by ion homeostasis) in an extraordinarily complex system. Several classes of candidate proteins are identified by statistical analysis and then examples are tested experimentally, which is great. Of course, in any global analysis one is likely to miss numerous non-global "exceptions" to the rule-in this case, for example, starvation survival proteins not linked to cell envelope integrity (or conversely, additional cell envelope proteins, like porins, that are not linked with starvation survival). I might suggest adding more discussion about this limitation. The strength of this analysis is that it identifies multiple proteins that are confirmed to modulate survival, but (of course) it is difficult to say with certainty that this class of proteins is sufficient or even dominant, though the work is suggestive of both. Again, this is not a major criticism and could be addressed with a brief discussion of potential caveats.

-- In a similar spirit, I might suggest a short discussion of whether the available data sets, and the corresponding ensemble of perturbations (on which the statistical analysis is based), may bias the search for candidate proteins in one direction or the other. In addition, are there limitations to keep in mind with the regularization scheme used to compare data across datasets? This is a notoriously difficult task, and while I find the approach solid, the manuscript might benefit from more discussion of the limitations and potential caveats.

Very Minor:
-- define GO first time it is used in main text
-- line 189: limiting --> limiting for

Response to reviewers

We thank all reviewers for their careful review of our work and their valuable suggestions. We are glad to hear that all reviewers appreciated our efforts, and we hope that we could sufficiently address their comments in our revised manuscript.

We answer to the specific points raised by the reviewers in the point-to-point response below. In addition, we performed two additional experiments prompted by changes requested by the reviewers, that, we believe, further strengthen our paper. Details on the new experiments are specified at the end of this document.

Reviewer #1:

Overall comment:
I have read the manuscript entitled "Adaptation of bacterial proteome reveals a key role of the cell envelope in starvation survival" authored by Schink et al. This is an interesting study that analyzes multiple protein abundance datasets across conditions and identified membrane-related genes play a key role to prevent cell death. The authors studied the trade-offs between fast growth and long survival and ranked more than 2000 E. coli proteins that are related to survival genes. Interestingly, they found the most important survival genes are playing roles in the maintenance of envelope integrity during starvation. These discoveries provide growing evidence of how the outer membrane is essential for the survival of non-sporulating bacteria. However, this manuscript needs significant work, specifically around the methods utilized, addressing the specific comments outlined below.

We thank the reviewer for the careful consideration of our work. We hope that the revised version sufficiently clarifies the methods that were used. To this end we carefully revised the text (pages 5-6), added a new Box that explains the methodology, updated figure captions, expanded the methods section, provided all necessary raw data and Jupyter Notebooks that allow reproducing our results with ease.

Major comments:
1. CACe-score is not a very clear name for this quantity – and it is not explicitly defined in the Methods, only in Figure S1. Recommend making sure that this specific term appears somewhere in the methods, and recommend changing the name of this score to something less unwieldy. Possibility: “Survival score”. The name for this score doesn’t have to precisely capture the underlying mathematical procedure used to compute it, as long as that procedure is very clear in the Methods and the term is defined explicitly there.

We like the idea of calling the score ‘Survival score’ and we implemented it in the manuscript. To improve the definition of the score, we have 1) improved presentation and description of the score in the manuscript, including a new Box and 2) uploaded commented code to derive the score from the raw data (see also next point).

2. The authors should carefully provide supplementary files to include raw data (e.g., protein abundance in the investigated conditions). The current pdf for review only has scattered tables, which are not readable. I found them from Biorxiv (10.1101/2022.05.18.492425) but it seems that only processed data (i.e., averaged values without error ranges) is available and raw data is still missing.

We apologize for any inconveniences in accessing the tables and we agree that raw data should be easily accessible. Initially we had only referenced the repositories from where we had downloaded the raw mass spectrometry data and detailed the processing in the methods. We now provide a Github repository (https://github.com/ammarcsj/ecoli_survival_scoring), which contains all the data and mappings used for the bioinformatics analyses along with files describing the contents of the directory along with documentation ('readme' files). To facilitate reproducibility, we have re-implemented the generation of the Survival Score from raw data using openly accessible Jupyter Notebooks, which we provide together with the data.

In the re-implementation, we have slightly modified our scoring procedure to make it more consistent and more easily understandable. The correlation of the old and the new Survival score is around 0.97 and the main conclusions are identical. We updated all tables to the new score and updated Figure 1C, which is affected by the change. To avoid scattered tables, we summarized the individual excel tables provided in the first submission into one supplemental table with improved presentation.

3. Since method and raw data were not sufficiently provided, it is difficult to understand how genes were chosen for testing in Figure 2. I see that some genes have high positive CACe-score values even if they have N/A or negative values in some conditions. And why do they fluctuate depending on conditions? Also, some of the tested genes have a vCACe-score value of nearly zero (e.g., bamE). Please elaborate on how the major finding is associated with the main analysis.

Testing genes was carried out by considering the Survival scores as well as our insights on the enriched processes associated as well as availability of knockouts in the Keio collection. In general, we chose Genes with high Survival Scores for testing, with some exemptions as stated below. This is also reflected in our list of Knockouts, which in general have relatively high Survival scores. In addition, to get good coverage of potentially interesting processes, we did not blindly follow the ordering of the Survival Score (which is subject to some inherent noise), but tried to pick genes, which are representative of different processes in Stress Response, Catabolism, or Envelope. This is also why there are some genes with lower Survival score present in the data, for example BamE.

In the manuscript, we now explicitly state that the choice of knockouts in the cell envelope, shown in Fig. 2B, was independent of their survival scores (page 8, lines 196-200):

> Motivated by this finding, we tested more cell envelope proteins, independent of their Survival scores, and found that starvation-survival is highly sensitive to knock-outs across different functionalities of the cell envelope (Fig. 2B), from outer membrane protein folding (BamE & Skp), cell wall synthesis and modification (Prc, NlpI, DolP & NlpD), phospholipid recycling (MlaA & MlaC) to cell envelope regulators (CpxA & RseA).

Concerning the NA/small/negative values: The Survival score is calculated by summarizing the Z-values of the individual CARLS conditions. Ideally, we have a well-defined Z-value in each of the conditions. Unfortunately, mass spectrometry data suffers from many missing values. This means, if we would constrain our dataset to only include genes with fully measured CARLS, less than 30% of the measured genes would be accessible to our analysis. Therefore we allow also incomplete CARLS measurements and summarize over the available values in case we have missing values. For the main enrichment analysis, we only used genes, which had at least 3 out of the 5 CARLS conditions measured, which we deemed a good compromise between completeness and comprehensiveness.

It can also happen that the Survival Score has still a high positive value even though one or even more of the conditions have negative values. In this case, the remaining conditions must be positive and have stronger values than the conditions with negative values. This should mostly happen when the values of the conditions are mildly negative, so the Z-values are not too strong. A not to strong Z-value means there is only a weak indication of the regulation going into the direction and this can be “overruled” by counter-evidence from the other conditions.

We have now added some further discussion of these and further aspects of the score in the main text and in the discussion section:

> Page 5, lines 112-114 (Results): “We collect these scores over the variety of conditions and data sets and iteratively merge the scores, while correcting for statistical dependencies and ignoring missing values […].”

> Page 6, lines 142-144 (Results): “Finally, we used Survival scores of all proteins measured in at least 3 of the 5 CARLS conditions to identify gene ontology (GO) processes and cellular compartments that contain proteins with significantly larger Survival scores than average.”

> Page 10, lines 282-284 (Discussion): “In order to retain the information available in the data set, we constructed our scores in such a way, that we omit missing values in the survival score, which leads to larger contributions of the available scores to the overall score.”

4. Similarly, the major conclusions are based on the death rate under different conditions. Please elaborate on the method about how to calculate the death rate was calculated in the Method section.

We expanded the Methods section on ‘Viability measurements’ with an explanation of how death rates were measured and renamed the section ‘Death rate measurements’.

5. The presence/absence of the "O" in "CARLOS" is confusing throughout. For example, in Figure S1, the top-right header implies that "O" is included in the CACe-score, while the histograms in the bottom center imply that it is not (which indeed it isn't). Consider eliminating "O" from the acronym and treating it as more of an "add-on" than an integral part of the survival score.

We agree with the reviewer the presence/absence of the ‘O’ condition can be confusing. While drafting the paper we in fact initially named the conditions ‘CARLS’. However, with ‘CARLS’ would omit the ‘O’ condition, which is an integral part of the growth perturbations, because it allows us to identify that the proteins of interest should be ‘beneficial’ rather than ‘harmful’.

There is no perfect solution. Because the acronym focuses on the growth perturbations we decided to stick with the ‘CARLOS’ acronym. We removed the ‘O’ when unnecessary and improved the text at various positions in the manuscript to alleviate the confusion. Fig. S1 quoted by the reviewer is now replaced with a Box. In the Box the ‘O’ is not mentioned anymore.

Minor comments:
Abstract: "...to narrow down the set of proteins that..."
Abstract: "...are related to the cell envelope..."
Introduction: "...the only energy source for heterotrophic..."
Introduction: "number of active processes without losing their viability" (remove hyphen)

Fixed.

Results: Figure 1B: 0 to max range in the color bar - what is max? Difficult to interpret 2-D histograms if they are all scaled differently. And it is not also clear what each gray square means and how the value was calculated. In the caption of Fig 1, some of the citations are referring to Fig. S3, not Fig. S2.

The grey squared show a 2D histogram, of all proteins were assigned Z-scores in both respective conditions. The colored 1D histograms shown on the sides of the plot correspond to all proteins that were assigned a Z-score in the respective condition. Both 1D and 2D histograms are normalized to their maximum value. While we agree that a normalization makes it harder to interpret the data, we believe that the normalization is necessary as the total number of proteins that are scored in CARLOS conditions varies depending on the quality of the data set.

We provide an improved explanation in the figure caption and main text, and we fixed the reference to Fig. S2. Changes include:

> Page 5, lines 118-119: "Histograms of these merged Z-values on the condition level (step 3) are shown as colored 1D histograms in Fig. 1B, normalized to the highest value."

> Page 5, lines 124-125: "Next, we compared Z-values of the conditions in a pair-wise manner by plotting 2D histograms of Z-values detected in both respective conditions (Fig. 1B, bottom left of matrix)."

Results: Figure 1C: FDR - is this just p-value? FDR is the threshold set for p-value correction, but I'm not sure that the corrected p-value itself can be interpreted as the actual false discovery rate. Consider re-labeling this column to "corrected p-value" or similar.

In Figure 1C, we report the adjusted p-values after Benjamini-Hochberg correction. The reported value should represent the highest threshold of type-I-errors (i.e. false discoveries in the set of significant hits) which can be chosen such that the null hypothesis can still be rejected. If we report an FDR of 0.01, this means that the underlying p-value must be just small enough to pass the Benjamini-Hochberg step-up procedure at FDR level 0.01. To avoid ambiguities, we have now specified the FDR procedure in the figure caption.

Results: "proteome changes in the remaining CARLS conditions are partially independent of each other, as evidenced by a significant fraction of Z-values being anticorrelated in each comparison" - what statistical test was used to determine the significance of the anti-correlated Z-values?

What we meant to say is that when comparing two perturbations, parts of the proteome are correlated, and parts are anti-correlated.

> Page 5, lines 125-129: "By classifying proteins that score above $|Z| > 1.28$, which corresponds to $p < 0.1$, as significantly correlated or anti-correlated, depending on the direction, we confirmed that proteome changes across different conditions are partially orthogonal to each other (Fig. 1B, top right of matrix). This means that each condition has additional information that can help us narrow down the target set of proteins."

The statistical power comes from the Z-values, which are the results of a statistical test, based on the MS-EmpiRe procedure

Results: "We next wanted to test if any..."
Results: "...in anaerobic conditions, where oxidative damage should be absent" (remove "the rate")
Results: "and up to 23% for bacteria..." (remove "a staggering")
Results: "...belonging to stress response and oxidative damage, showed little..." (add comma)
Figure 2A, please indicate both growth stage and medium for each condition.

Fixed all the above.

Figure S2: "less than X" - what is X?

We forgot to insert the number. The threshold is at 1.28.

Figure S3: y-axis should be 0 to 1 for the cumulative frequency label (similar to Figure 1C). The 0 and 2.5 colored lines are not clearly visible, making interpretation difficult.

Fixed.

Figure S5: legend says proteins with FC higher than 2 are red, but plot has red color for proteins with FC greater than 1

Figure EV4 (old Fig. S5) shows log2(FC), cut-off is at log2(2) = 1. Coloring is correct. Because a fold change of 2 is sometimes referred to as a 'one fold increase' (according to Wikipedia), we included a definition in the caption to prevent any confusion.

Figure S5: what does the bracket between 40C and pH 6 mean?

This was supposed to show that the p value of each comparison of the control with the three stresses is larger than 0.5. We replaced the bracket with individual comparisons, which are hopefully clearer.

Results: "...mechanical integrity of the cell envelope plays a causal role..." (remove "is")
Results: "supplementing starved culture with EDTA" - Figure 2D has DNP on the x-axis - which is correct?

Fixed. DNP is correct. We also performed the experiment with EDTA leading to similar results, hence the confusion.

It seems that a death rate is referred for both "cell" and "protein". Please differentiate them in the text and detail how they were calculated if not mentioned clearly.

In our paper, death rates are always extracted from starvation-survival curves. Viability is measured in colony forming units over time, plotted on a semi-log scale and the death rate is extracted from a linear fit. We either change the growth conditions prior to starvation (CARLOS conditions), the conditions during starvation ('osmo-balanced medium', Mg2+ or DNP variations), or we use mutants grown and starved in our reference conditions. Death rates are not supposed to refer to proteins.

Because the confusion came from an ill-defined ‘death rate’, we added additional explanations whenever the death rate of a new condition/perturbation was tested, e.g.

> Page 7, lines 164-165: “Similarly, adding antioxidants that prevent carbonylation during starvation did not decrease the death rate of starvation E. coli (Fig. S4B, glutathione, ascorbic acid and mercaptoethanol).”

> Page 7, lines 189-191: “In contrast, knockouts of most other genes, especially those belonging to stress response and oxidative damage, had little or no impact of death rates in starvation (Table S5), further corroborating that many of them are non-limiting in our conditions.”

Figure 1 panel A "Glucose minmal medium" should be "Glucose minimal medium";

Fixed.

Figure 1C, the author emphasized that several GO-processes were enriched, including "Response to oxidative stress" and "Response to stress". It seems that the "Response to stress" means non-oxidative stress. It is necessary to appropriately describe the "Response to stress" here.

We expanded our discussion around the GO-processes. We mention that the GO-processes are non-mutually exclusive, meaning that proteins can be part of several GO-processes:

> Page 6, lines 147-149: “Note that GO-processes are not mutually exclusive and can contain the same proteins, which is particularly true for ‘oxidative stress response’, which substantially overlaps with ‘response to stress’.”

Each growth condition has a corresponding death rate. Assuming that the authors tested the death rate under the glucose minimal conditions. It should be noted in the figure legend.

Fixed.

Please check and appropriately make the font italic. Here Line 140, Biselli et al., 2020; Line142, and other lines.
Line 163 "rseA" should be "RseA"
Line 178 "is plays a causal" should be "plays..."

Fixed.

Reviewer #2:

A better understanding cell death represents a unique opportunity in bacterial physiology. Previous work by the authors established key mechanisms underlying the death rate in E. coli K-12: 1. balance between maintenance rate and recycling yield sets the death rate, and 2. That there is a trade-off between growing faster and surviving longer. This study is a natural follow-up and addresses what the molecular determinants of adaptation are during this growth-death transition.

The authors compare mass spectrometry (MS) data for different growth perturbation experiments

(CARLOS) to obtain candidate proteins by scoring their correlation strength across datasets. Upon further annotation proteins involved in the cell envelope functions show a considerable increase in death rates. This is confirmed by a genetic screen where the knockouts display elevated death rates. Next, the involvement of these mutants in impairing cell wall integrity and prior knowledge of the effects of ion homeostasis on death leads the authors to hypothesize that the integrity of cell wall in general and membrane permeability, in particular, is crucial and is more specifically mediated by the cost of ion homeostasis.

To test this hypothesis they minimize ion diffusion across the membrane by using an osmo-balanced medium to find a decrease in death rate and that the growth-death dependence across different conditions is absent. If the cost of ion homeostasis determines membrane integrity, even for widely different investments into the cell envelope proteins for different conditions, we would expect similar death rates, hence the observation (Fig. 3). This supports their hypothesis and leads them to conclude that the growth-death rate dependence from Fig 1A follows from differences in cell envelope integrity under those conditions.

We feel that this finding is novel and represents a significant advance that the readers of MSB would be interested in. Overall, we recommend this work for publication after the authors respond to the questions and comments below.

We thank the reviewer for their careful consideration of our work. The major update of the paper that we performed in response to this review is that we included a Box to explain the methodology, which is accompanied by an improved main text, methods section and Jupyter Notebooks that allow the reader to reproduce our calculations. We hope that the manuscript is now easier to follow around the statistical scoring part.

Major points:

The manuscript was quite hard to follow around the statistical scoring part. I feel parts of Fig S2 should be included in the main text or some more time should be spent on explaining the entirety of Fig. 1. Another suggestion is to include an illustration that runs through calculations and scores in Fig. 1b by using an example for 1 protein.

We would like to thank the reviewers for highlighting this shortcoming of our work and proposing a nice solution. As suggested have now added a Box visualizing and explaining the methodology based on the distinct steps of the algorithm for an individual protein. Additionally, we provide Jupyter Notebooks via Github (https://github.com/ammarcsj/ecoli_survival_scoring), which implement the steps described in the Box.

Line 206: While Fig 3. is the culmination of this story, it does merit a more elaborate explanation, in that it was not obvious why the authors would expect the tradeoff to go away upon using an osmo-balanced medium. Maybe elaborating this inference could help make the expectation clearer.

We substantially expanded the text surrounding Fig. 3, including, but not limited to, the following change:

> Page 9, lines 242-254: “This medium reduces the death around 4-fold in standard glucose minimal medium conditions <u>due to a reduction in maintenance rate required for ion</u>

homeostasis (Schink et al., 2021). If changes in the membrane permeability were responsible for the modulation of death rate in Fig. 1, we predict that the osmo-balanced medium should remove the growth-death dependence. […] On the other hand, if the growth-death dependence is independent of permeability of ions, for example if the modulated proteins and processes are preventing some type of damage or are influencing nutrient recycling, then we would expect the growth-death dependence to remain in ‘osmo-balanced’ medium but be shifted to a lower death rate.”

How do the authors decide to wash the medium rather than let the nutrients deplete naturally? I am ok with the experiments being in some sense "unnatural", but I also feel that it would be good for the reader to be aware of which conclusions depend on this and which do not.

Good idea. We changed the text to:

> Page 4, lines 95-108: “We studied Escherichia coli K-12, first grown in single carbon source N+C+ minimal medium, followed by a wash and resuspension in medium with the carbon source missing. The washing step introduces bacteria suddenly to starvation and prevents their complete adaptation. As a result, during the ensuing starvation, viability decreases exponentially (Phaiboun et al., 2015; Schink et al., 2019), with death rate depending on the previous growth condition. E. coli grown on carbon substrates supporting faster growth die faster than those on poor carbon substrates (Biselli et al., 2020), see Fig. 1A ('blue’). Alternatively, letting E. coli adapt to stationary phase on glucose minimal medium results in a death rate similar to sudden starvation from very low growth rates (Fig. 1A, open symbols placed at growth rate zero).”

> Page 4, line 92-95: “To narrow down the search for the proteins that determine lifespan, we decided to use additional growth conditions that show different changes in proteome. We identified a total of six independent perturbations, which we collectively call ‘CARLOS conditions’ that affect proteome composition during growth and death rate after washing and resuspension in carbon-free medium.”

Minor points:

Introduction:

1st and 2nd para:
"This finite...": What does this refer to? Aren't both the aforementioned resources finite?
The first and second paragraphs can be demarcated better. "...But what determines..." could be shifted to the second paragraph.

Yes, both are finite. We changed the text to (page 3, line 57): “These finite energy sources […]”

Results:
Para 4: line 98: "...as evidenced by a significant...": Does anti-correlation imply independence?

Independence might not have been the right word to use here. We didn’t mean to say ‘statistically independent’, but rather that parts of the proteome response are correlated while others are anti-correlated. We rewrote the text to reflect that and replaced ‘independent’ with ‘partially orthogonal’:

Page 5, lines 124-129: “Next, we compared Z-values of the conditions in a pair-wise manner by plotting 2D histograms of Z-values detected in both respective conditions (Fig. 1B, bottom left of matrix). By classifying proteins that score above $|Z| > 1.28$, which corresponds to $p < 0.1$, as significantly correlated or anti-correlated, depending on the direction, we confirmed that proteome changes across different conditions are partially orthogonal of each other (Fig. 1B, top right of matrix). This means that each condition has additional information that can help us narrow down the target set of proteins.”

Grammatical errors/typos:

Line 17: set of proteins*
Line 116: wanted TO test
Line 126: rate oxidative?
Line 178: extra "is"
Line 196: Add citation
Line 207: reduce*
Line 243: stabilize

Fixed.

Reviewer #3:

The manuscript by Schink and colleagues investigates how bacteria reorganize the proteome to promote starvation survival. Using a set of 6 growth perturbations ("CARLOS" conditions, defined in Figure 1, drawing on previously published data) to generate an ensemble of proteome responses to nutrient limitations, they identify a set of candidate proteins linked with survival and lifespan-and in doing so evade the challenges often associated with this task, namely the fact that most physiological measures can be correlated with lifespan. To do so, they calculate a statistical score ("Z-value") that quantifies the probability that protein abundance is correlated (or anti-) with longer survival. They calculate regularized Z-values for each dataset and then aggregated Z-values per condition. When compared pairwise, the Z-values per condition show enough variation (e.g. sizable fraction of Z-values are anti-correlated, yet Z-values for "similar" perturbations are correlated) that they are assumed partially independent, which is the key to identifying a reduced set of candidate proteins linked with starvation survival. The authors then narrowed their focus, guided by experiments, to a set of cell envelope proteins, and they found that knocking out these envelope proteins-which span multiple functions (e.g. cell wall synthesis or cell envelope regulation)-significantly modulated starvation survival (porins are a notable exception). They complemented these findings by applying antimicrobials targeting the cell envelope, which they show kill the cells during starvation (a notable corroborating finding given that many antibiotics, such as beta-lactams, kill in a growth-rate dependent manner and would lose effectiveness in starvation conditions). Finally, they show that the death-rate dependence is eliminated in osmotically balanced media, suggesting (along with previous work) a connection to ion homeostasis.

Overall, I find the work interesting and timely. It draws on a clever use of previous data to identify target proteins linked with survival, and importantly, tests those predictions experimentally for a collection of proteins. The results are surprising given that the periplasm and outer membrane are typically considered to function primarily as barriers to entry (for, say, antimicrobials or other harmful

substances), and I therefore believe it would interest a broad range of readers. I have only the following minor comments for the author to consider:

-- The paper does a nice job of extracting simple principles (i.e. cell envelope integrity modulates starvation survival, perhaps driven by ion homeostasis) in an extraordinarily complex system. Several classes of candidate proteins are identified by statistical analysis and then examples are tested experimentally, which is great. Of course, in any global analysis one is likely to miss numerous non-global "exceptions" to the rule-in this case, for example, starvation survival proteins not linked to cell envelope integrity (or conversely, additional cell envelope proteins, like porins, that are not linked with starvation survival). I might suggest adding more discussion about this limitation. The strength of this analysis is that it identifies multiple proteins that are confirmed to modulate survival, but (of course) it is difficult to say with certainty that this class of proteins is sufficient or even dominant, though the work is suggestive of both. Again, this is not a major criticism and could be addressed with a brief discussion of potential caveats.

-- In a similar spirit, I might suggest a short discussion of whether the available data sets, and the corresponding ensemble of perturbations (on which the statistical analysis is based), may bias the search for candidate proteins in one direction or the other. In addition, are there limitations to keep in mind with the regularization scheme used to compare data across datasets? This is a notoriously difficult task, and while I find the approach solid, the manuscript might benefit from more discussion of the limitations and potential caveats.

The above points are important suggestions by the reviewer. In the discussion section of the manuscript, we now include a section titles ‘Limitations of the study’ in which we lay out the most important pitfalls and biases of our analysis, including 1. Exceptions to the rule, 2. Proteins not connected to the cell envelope, 3. Bias due to measurement limitations and 4. Bias towards Carbon limitation.

The section is long, so to save space we do not copy paste it here. You can find it in the beginning of the Discussion section, starting on page 10, line 264.

Very Minor:
-- define GO first time it is used in main text
-- line 189: limiting --> limiting for

Fixed.

New Additional Experiments

Prompted by changes proposed by the reviewers, we also improved the paper on two aspects with new experiments:

1. We improved our analysis of the stress response and brought it in-line with the existing literature. We probed the death rate two stains: a knock-out of *rssB* and a ‘fixed’ version of RpoS. RssB targets RpoS for degradation. This strain was previously reported to survive better than wild-type by Fontaine et al, which we confirmed in our system (Fig. EV4E). The ‘fixed-rpoS’

version removes an amber stop codon in our E. coli strain, which results in an increased abundance of rpoS dependent genes (Mori et al). This fixed-rpoS strain did not decrease death rate. We believe that our conclusion that the stress response it not the major driver of the growth-death relation is still correct and detail this in the main text. The new experiment allows the manuscript to be better integrated to the current knowledge in the field.

2. Previously Fig. 3 which showed death rate in ‘osmo-balanced’ medium for a subset of the CARLOS condition: rich media (L), various carbon minimal media (C) and stationary phase adapted (S) cultures. The revised version includes the missing conditions: anabolic limitation (A), LacZ overexpression (O) and ribosome limitation (R). In addition, we noticed a mistake in the fit of two sets of experiments that we corrected (acetate and glucose reference medium). Because with the new set of data there is still no correlation of death rate with growth rate in the ‘osmo-balanced’ medium, there is no change in the interpretation of our data.

Thank you for sending us your revised manuscript. We have now received the comments from the two referees who agreed to re-review your manuscript. As you will see below, the referees are satisfied with the modifications and think the study is now suitable for publication.

Before we can formally accept your manuscript, we would ask you to address the following editorial-level issues:

Reviewer #1:

The authors have substantially improved the manuscript and addressed the Reviewer critiques.

Reviewer #3:

I think the paper has been significantly improved thanks to a thorough discussion of potential limitations. I have no further suggestions.

2nd Revision - Editorial Decision **19th Oct 2022**

Thank you again for sending us your revised manuscript. We are now satisfied with the modifications made and I am pleased to inform you that your paper has been accepted for publication.

EMBO Press Author Checklist

Corresponding Author Name: Markus Basan	
Journal Submitted to: Molecular Systems Biology	
Manuscript Number: MSB-2022-11160	

USEFUL LINKS FOR COMPLETING THIS FORM
The EMBO Journal - Author Guidelines
EMBO Reports - Author Guidelines
Molecular Systems Biology - Author Guidelines
EMBO Molecular Medicine - Author Guidelines

Reporting Checklist for Life Science Articles (updated January
This checklist is adapted from Materials Design Analysis Reporting (MDAR) Checklist for Authors. MDAR establishes a minimum set of requirements in transparent reporting in the life sciences (see Statement of Task: 10.31222/osf.io/9sm4x). Please follow the journal's guidelines in preparing your
Please note that a copy of this checklist will be published alongside your article.

Abridged guidelines for figures
1. Data
The data shown in figures should satisfy the following conditions:
- the data were obtained and processed according to the field’s best practice and are presented to reflect the results of the experiments in an accurate and unbiased manner.
- ideally, figure panels should include only measurements that are directly comparable to each other and obtained with the same assay.
- plots include clearly labeled error bars for independent experiments and sample sizes. Unless justified, error bars should not be shown for technical
- if n<5, the individual data points from each experiment should be plotted. Any statistical test employed should be justified.
- Source Data should be included to report the data underlying figures according to the guidelines set out in the authorship guidelines on Data

2. Captions
Each figure caption should contain the following information, for each panel where they are relevant:
- a specification of the experimental system investigated (eg cell line, species name).
- the assay(s) and method(s) used to carry out the reported observations and measurements.
- an explicit mention of the biological and chemical entity(ies) that are being measured.
- an explicit mention of the biological and chemical entity(ies) that are altered/varied/perturbed in a controlled manner.
- the exact sample size (n) for each experimental group/condition, given as a number, not a range;
- a description of the sample collection allowing the reader to understand whether the samples represent technical or biological replicates (including how many animals, litters, cultures, etc.).
- a statement of how many times the experiment shown was independently replicated in the laboratory.
- definitions of statistical methods and measures:
  - common tests, such as t-test (please specify whether paired vs. unpaired), simple χ2 tests, Wilcoxon and Mann-Whitney tests, can be unambiguously identified by name only, but more complex techniques should be described in the methods section;
  - are tests one-sided or two-sided?
  - are there adjustments for multiple comparisons?
  - exact statistical test results, e.g., P values = x but not P values < x;
  - definition of ‘center values’ as median or average;
  - definition of error bars as s.d. or s.e.m.

Please complete ALL of the questions below.
Select "Not Applicable" only when the requested information is not relevant for your study.

Materials

Newly Created Materials	Information included in the manuscript?	In which section is the information available? (Reagents and Tools Table, Materials and Methods, Figures, Data Availability Section)
New materials and reagents need to be available; do any restrictions apply?	Not Applicable	

Antibodies	Information included in the manuscript?	In which section is the information available? (Reagents and Tools Table, Materials and Methods, Figures, Data Availability Section)
For **antibodies** provide the following information:<br>- Commercial antibodies: RRID (if possible) or supplier name, catalogue number and or/clone number<br>- Non-commercial: RRID or citation	Not Applicable	

DNA and RNA sequences	Information included in the manuscript?	In which section is the information available? (Reagents and Tools Table, Materials and Methods, Figures, Data Availability Section)
**Short novel DNA or RNA including primers, probes**: provide the sequences.	Not Applicable	

Cell materials	Information included in the manuscript?	In which section is the information available? (Reagents and Tools Table, Materials and Methods, Figures, Data Availability Section)
**Cell lines:** Provide species information, strain. Provide accession number in repository **OR** supplier name, catalog number, clone number, and/**OR** RRID.	Not Applicable	
**Primary cultures:** Provide species, strain, sex of origin, genetic modification status.	Not Applicable	
Report if the cell lines were recently **authenticated** (e.g., by STR profiling) and tested for mycoplasma contamination.	Not Applicable	

Experimental animals	Information included in the manuscript?	In which section is the information available? (Reagents and Tools Table, Materials and Methods, Figures, Data Availability Section)
**Laboratory animals or Model organisms:** Provide species, strain, sex, age, genetic modification status. Provide accession number in repository **OR** supplier name, catalog number, clone number, **OR** RRID.	Not Applicable	
**Animal observed in or captured from the field:** Provide species, sex, and age where possible**.**	Not Applicable	
Please detail **housing and husbandry conditions**.	Not Applicable	

Plants and microbes	Information included in the manuscript?	In which section is the information available? (Reagents and Tools Table, Materials and Methods, Figures, Data Availability Section)
**Plants:** provide species and strain, ecotype and cultivar where relevant, unique accession number if available, and source (including location for collected wild specimens).	Not Applicable	
**Microbes:** provide species and strain, unique accession number if available, and source.	Yes	In Materials and Methods subsection 'Strains'

Human research participants	Information included in the manuscript?	In which section is the information available? (Reagents and Tools Table, Materials and Methods, Figures, Data Availability Section)
If collected and within the bounds of privacy constraints report on age, sex and gender or ethnicity for all study participants.	Not Applicable	

Core facilities	Information included in the manuscript?	In which section is the information available? (Reagents and Tools Table, Materials and Methods, Figures, Data Availability Section)
If your work benefited from core facilities, was their service mentioned in the acknowledgments section?	Not Applicable	

Design

Study protocol	Information included in the manuscript?	In which section is the information available? (Reagents and Tools Table, Materials and Methods, Figures, Data Availability Section)
If study protocol has been **pre-registered, provide DOI in the manuscript**. For clinical trials, provide the trial registration number **OR** cite DOI.	Not Applicable	
Report the **clinical trial registration number** (at ClinicalTrials.gov or equivalent), where applicable.	Not Applicable	

Laboratory protocol	Information included in the manuscript?	In which section is the information available? (Reagents and Tools Table, Materials and Methods, Figures, Data Availability Section)
Provide DOI OR other citation details if **external detailed step-by-step protocols** are available.	Not Applicable	

Experimental study design and statistics	Information included in the manuscript?	In which section is the information available? (Reagents and Tools Table, Materials and Methods, Figures, Data Availability Section)
Include a statement about **sample size** estimate even if no statistical methods were used.	Not Applicable	
Were any steps taken to minimize the effects of subjective bias when allocating animals/samples to treatment (e.g. **randomization procedure**)? If yes, have they been described?	Not Applicable	
Include a statement about **blinding** even if no blinding was done.	Not Applicable	
Describe **inclusion/exclusion criteria** if samples or animals were excluded from the analysis. Were the criteria pre-established?<br><br>If sample or data points were omitted from analysis, report if this was due to attrition or intentional exclusion and provide justification.	Not Applicable	
For every figure, are **statistical tests** justified as appropriate? Do the data meet the assumptions of the tests (e.g., normal distribution)? Describe any methods used to assess it. Is there an estimate of variation within each group of data? Is the variance similar between the groups that are being statistically compared?	Yes	Statistical tests are described in the figure captions and Material and Methods

Sample definition and in-laboratory replication	Information included in the manuscript?	In which section is the information available? (Reagents and Tools Table, Materials and Methods, Figures, Data Availability Section)
In the figure legends: state number of times the experiment was **replicated** in laboratory.	Yes	Number of relpicates are written in the respective figure captions
In the figure legends: define whether data describe **technical or biological replicates**.	Yes	

Ethics

Ethics	Information included in the manuscript?	In which section is the information available? (Reagents and Tools Table, Materials and Methods, Figures, Data Availability Section)
Studies involving **human participants**: State details of **authority granting ethics approval** (IRB or equivalent committee(s), provide reference number for approval.	Not Applicable	
Studies involving **human participants**: Include a statement confirming that **informed consent** was obtained from all subjects and that the experiments conformed to the principles set out in the WMA Declaration of Helsinki and the Department of Health and Human Services Belmont Report.	Not Applicable	
Studies involving **human participants:** For publication of **patient photos**, include a statement confirming that consent to publish was obtained.	Not Applicable	
Studies involving experimental **animals**: State details of **authority granting ethics approva**l (IRB or equivalent committee(s), provide reference number for approval. Include a statement of compliance with ethical regulations.	Not Applicable	
Studies involving **specimen and field samples:** State if relevant **permits** obtained, provide details of authority approving study; if none were required, explain why.	Not Applicable	

Dual Use Research of Concern (DURC)	Information included in the manuscript?	In which section is the information available? (Reagents and Tools Table, Materials and Methods, Figures, Data Availability Section)
Could your study fall under dual use research restrictions? Please check biosecurity documents and list of **select agents and toxins** (CDC): https://www.selectagents.gov/sat/list.htm	Not Applicable	
If you used a select agent, is the security level of the lab appropriate and reported in the manuscript?	Not Applicable	
If a study is subject to dual use research of concern regulations, is the name of the **authority granting approval and reference number** for the regulatory approval provided in the manuscript?	Not Applicable	

Reporting

The MDAR framework recommends adoption of discipline-specific guidelines, established and endorsed through community initiatives. Journals have their own policy about requiring specific guidelines and recommendations to complement MDAR.

Adherence to community standards	Information included in the manuscript?	In which section is the information available? (Reagents and Tools Table, Materials and Methods, Figures, Data Availability Section)
State if relevant guidelines or checklists (e.g., **ICMJE, MIBBI, ARRIVE, PRISMA**) have been followed or provided.	Not Applicable	
For **tumor marker prognostic studies,** we recommend that you follow the **REMARK** reporting guidelines (see link list at top right). See author guidelines, under 'Reporting Guidelines'. Please confirm you have followed these guidelines.	Not Applicable	
For **phase II and III randomized controlled trials**, please refer to the **CONSORT** flow diagram (see link list at top right) and submit the CONSORT checklist (see link list at top right) with your submission. See author guidelines, under 'Reporting Guidelines'. Please confirm you have submitted this list.	Not Applicable	

Data Availability

Data availability	Information included in the manuscript?	In which section is the information available? (Reagents and Tools Table, Materials and Methods, Figures, Data Availability Section)
Have **primary datasets** been deposited according to the journal's guidelines (see 'Data Deposition' section) and the respective accession numbers provided in the Data Availability Section?	Not Applicable	
Were **human clinical and genomic datasets** deposited in a public access-controlled repository in accordance to ethical obligations to the patients and to the applicable consent agreement?	Not Applicable	
Are **computational models** that are central and integral to a study available without restrictions in a machine-readable form? Were the relevant accession numbers or links provided?	Yes	Links are provided in the Data Availability section.
If publicly available data were reused, provide the respective **data citations in the reference list**.	Yes	Data references
